# Determinants of Severe Financial Distress in U.S. Acute Care Hospitals: A National Longitudinal Study

**DOI:** 10.3390/healthcare14030366

**Published:** 2026-01-31

**Authors:** James R. Langabeer, Francine R. Vega, Audrey Sarah Cohen, Tiffany Champagne-Langabeer, Andrea J. Yatsco, Karima Lalani

**Affiliations:** 1 School of Biomedical Informatics, The University of Texas Health Science Center at Houston, Houston, TX 77030, USA; francine.r.vega@uth.tmc.edu (F.R.V.);; 2 School of Public Health, The University of Washington, Seattle, WA 98195, USA; klalani@uw.edu

**Keywords:** financial distress, hospital, acute care, profitability, Z-score

## Abstract

**Highlights:**

**What are the main findings?**

- Nearly one in five U.S. short-term acute care hospitals are in severe financial distress, with rising debt and declining margins placing more than 566 facilities at heightened risk of closure or bankruptcy.
- Larger, urban hospitals with larger debt ratios were more likely to be distressed.

**What are the implications of the main findings?**

- Hospital financial distress at this scale signals a growing risk of service reductions and closures that could undermine regional healthcare capacity and limit access in many communities.

**Abstract:**

**Background**: Financial sustainability remains a central challenge for U.S. hospitals as rising operating costs, shifting federal reimbursement, and policy uncertainty intensify economic pressures. This study estimates the prevalence and recent changes in financial distress among U.S. short-term acute care hospitals. **Methods**: We conducted a national longitudinal analysis of all U.S. short-term acute care hospitals from 2021 to 2023 using financial and operational data from Medicare cost reports linked with community-level data from the American Community Survey. Financial distress was measured using the Altman Z-score, with severe distress defined as Z ≤ 1.8. Logistic regression models were used to identify organizational, operational, and market characteristics associated with distress. **Results**: The proportion of hospitals classified as severely financially distressed increased from 18.6% in 2021 to 22.0% in 2023. Operating margins and returns on assets declined significantly over the study period, while mean Z-scores showed a modest but non-significant downward trend. In adjusted models, urban hospitals had higher odds of distress (OR 1.27, 95% CI 1.15–1.40, *p* < 0.001), as did hospitals with longer average lengths of stay (OR 1.07 per day, 95% CI 1.04–1.09, *p* < 0.001) and higher debt-to-equity ratios (OR 1.05 per unit, 95% CI 1.05–1.06, *p* < 0.001). Higher occupancy rates were protective (OR 0.31, 95% CI 0.25–0.40, *p* < 0.001). Larger market population was also associated with increased distress risk (OR 1.61, 95% CI 1.21–2.14, *p* = 0.001), while other market characteristics were not significant. **Conclusions**: Financial distress remains widespread and appears to be increasing among U.S. acute care hospitals. Operational efficiency, capital structure, and local market scale are key drivers of financial vulnerability, highlighting the need for targeted strategies to strengthen hospital resilience and preserve access to essential acute care services.

## 1. Introduction

Hospital financial performance remains a critical concern in healthcare, as organizations lacking adequate financial resources are unable to fulfill their mission of providing high-quality patient care. The healthcare industry has explicitly been cited as operating with some of the lowest median operating margins across sectors [1]. When hospitals consistently generate revenues below their expenses, financial strain emerges, as losses are absorbed through reductions in equity or fund balance, which ultimately diminishes the organization’s overall economic value [2].

Financial distress arises when a hospital struggles to meet its financial obligations to employees, vendors, creditors, or investors [3]. Persistent distress can lead to severe consequences, including organizational closure, acquisition, or bankruptcy [2]. The capacity to identify and potentially mitigate hospitals at risk of severe financial distress represents a critical yet underexplored area within health systems research.

Hospital closures have profound implications for the communities they serve [4]. Beyond the economic disruption caused by the loss of substantial payrolls supporting both clinical and non-clinical staff, closures also reduce access to essential healthcare services. In many cases, affected populations are left with limited or no viable alternatives for care [5]. In other studies, surviving hospitals reduced both the number of service lines offered and their service durations, which negatively impacts the surrounding community [6].

While hospital financial stability is a global concern, the drivers and policy levers differ across health system models [7]. A complex interaction of macroeconomic pressures, organizational structures, geographic disparities, and policy decisions shapes the financial sustainability of U.S. hospitals. Hospital financial performance is shaped not only by internal operations and capital structure, but also by coercive pressures through regulation and reimbursement rules, normative pressures from accreditation and professional standards, and mimetic pressures from competitive benchmarking under uncertainty and resource limitations [8,9,10]. These institutional pressures influence cost structures, service configurations, resource availability, and the degree of strategic flexibility hospitals have when responding to unprecedented economic shocks and evolving policy environments [11]. The COVID-19 pandemic and its aftermath, inflation, labor shortages, and declining reimbursement rates and policy changes have intensified existing vulnerabilities that impact financial distress [12,13,14].

Much of the research on financial distress has focused on rural markets, or small areas outside of urban cities with low population density [15]. Another study found that nearly 11% of rural hospitals in the U.S. were at high risk of closure [16]. Carroll and colleagues [4] examined data from 2010 to 2018 and found that, among hospitals that were unprofitable at baseline, nearly 7% closed and another 17% merged with another system or were acquired. When rural hospitals closed, hospital prices typically increased in that same region [17].

Another study examined 310 acute care hospitals in Texas and found that 16.1% were identified as financially distressed [18]. These hospitals were smaller, had lower patient acuity, and generated less outpatient revenue. System affiliation emerged as a protective factor, offering an operational and financial buffer. High-performing hospitals had greater market share and lower teaching intensity, suggesting that strategic growth and operational efficiency are critical to financial sustainability. A similar study found that major teaching hospitals, those with medical residency training programs, were shown to benefit from a larger proportion of outpatient care and larger system affiliation [19].

Geographical factors of the surrounding external market have been shown to be associated with strategic performance of organizations. One theory suggests that spatial forces help support a competitive advantage, specifically through the impact on strategic location decisions for new sites [20]. Other theories emphasize the concept of embeddedness, where organizational strategies are formed based on relationships with the economic, social, and political environment [21].

Management strategies play a role in how hospitals compete [22]. Douglas & Ryman demonstrated that hospitals could build a unique competitive advantage through choices of their market structure and firm-level competencies [23]. Why some hospitals outperform others has been linked to several theories, such as more extensive strategic resources and capabilities, as outlined in a resource-based view [24,25]. Solid, optimistic leadership and decision-making could also play a significant role in identifying a more attractive market niche and competitive advantage. Environmental forces, such as competition and geographic characteristics of the region served, likewise have been shown to influence financial viability [26,27].

Given the complexity of the relationship between hospital performance and internal and external factors, there remains a gap in the literature. We conceptualize hospital financial distress as an outcome shaped by internal financial conditions captured in standardized distress metrics such as liquidity, leverage and operating performance, and external, structural and community level context that influences revenue and stability cost (e.g., local socioeconomic conditions, rural, and community health factors). The objective of this study is to quantify the current state of financial distress among all U.S. acute care hospitals longitudinally and to examine how both organizational factors and geographic determinants influence the financial sustainability of U.S. hospitals.

## 2. Materials and Methods

### 2.1. Study Design

This observational study is a longitudinal analysis of all short-term acute-care hospitals operating in the United States from 2021 to 2023. Hospital financial and operating data were obtained through a subscription from the American Hospital Directory, based on extracts of the Centers for Medicare and Medicaid hospital cost reports, which are filed annually. Community-level data were extracted from the American Community Survey [15] (US Census). All data sources are publicly available and were accessed via direct download.

### 2.2. Measures

#### 2.2.1. Outcome Measure Primary Dependent Variable Severe Financial Distress

The primary dependent variable severe financial distress was defined using the Altman Z-score (non-manufacturing specifications). The Altman Z-score is used to predict financial distress across numerous industries, including healthcare [28,29]. We classified hospitals as severely distressed when Z ≤1.8. The Altman Z-score is a linear combination of multiple financial ratios that collectively reflect a hospital’s liquidity, profitability, and leverage [30,31]. The formula for non-manufacturing industries is expressed as follows:

Z = 6.56X1 + 3.26X2 + 6.72X3 + 1.05X4

In this formula, X1 represents net working capital divided by total assets. X2 = retained earnings divided by total assets; X3 represents total earnings before interest and tax divided by total assets, and X4 represents total equity or fund balance divided by total liabilities.

Z-scores were calculated separately for each hospital-year observation using fiscal-year cost report data. We analyzed the Z-score both as a continuous measure and as a binary indicator of severe financial distress. The logistic regression used this binary measure, applying a cutoff score of 1.8 or below to indicate severe financial distress, typically indicative of bankruptcy within two years [29]. A score between 1.81 and 3.0 would represent a zone of caution, while any score greater than 3.0 would indicate solid financial condition.

To address extreme values driven by ratio instability and division-by-zero for hospitals with no limited financial data, we first removed invalid z-scores and then excluded extreme z-score outliers. These outliers were identified through the exploratory distributional diagnostics stem-and-leaf method. Observations with Z-scores ≤−8.3 and ≥19.9 were excluded, yielding 7900 hospital-year observations.

#### 2.2.2. Hospital Organizational and Operational Characteristics

Hospital-level characteristics included size, organizational form, location, operational capacity, and financial structure. Size was measured as staffed beds. Organizational form was captured using ownership/control type (government, voluntary non-profit, and proprietary). Hospital location was captured using an urban/rural indicator; zip code was used to link market characteristics to hospitals. Operational capacity was measured using occupancy rate, calculated as inpatient days divided by available bed-days and average length of stay (ALOS). Financial structure was measured using the debt-to-equity ratio calculated as total liabilities divided by total equity.

#### 2.2.3. Community/Market Characteristics and Linkage

Community-level measures were linked to hospitals using the hospital zip code and included median household income, uninsured prevalence, population size, average age and obesity prevalence. Zip code linkage was used as a proxy for local market context as patient-origin or catchment-area data were not available.

### 2.3. Study Sample Dataset Construction

The analytic dataset was structured at the hospital-year level, with each observation representing one short-term acute hospital’s fiscal-year record in FY 2021, FY 2022, or FY 2023. The Altman Z-score and all hospital-level covariates were calculated separately for each hospital-year. The resulting dataset was unbalanced, as some hospitals did not contribute observations in all three years due to either incomplete reporting of required cost report elements and/or entry/exit from the reporting frame within the study window. Hospital-year observations missing required elements were excluded from analysis using a complete-case approach for model estimation. We examined the distribution of repeated measures by calculating the number of years contributed per hospital, reported alongside year-specific descripted characteristics. The dataset does not include a direct indicator of closure, bankruptcy, or suspension; therefore, cessation of operations is not separately coded and is reflected indirectly through noncontribution of subsequent hospital-year observations.

### 2.4. Statistical Analyses

We used multiple statistical analyses in this study. First, we calculated the Altman Z-score described above. Second, we developed a logistic regression model using a Z-score cutoff of 1.8, with 1 = distressed facility and 0 = non-distressed. We calculated descriptive statistics for all variables. Measures of financial performance we assessed included the Z-score, return on assets (ROA), and operating margin. We also analyzed structural indicators, including variables for total staffed beds (reflecting size), ownership or control type (i.e., government, non-profit, or proprietary) and two measures of hospital efficiency (i.e., occupancy rate, average length of stay). We operationalized efficiency using two widely used operational efficiency indicators: occupancy rate and average length of stay (ALOS). The occupancy rate reflects capacity utilization (inpatient days relative to available bed-days), and ALOS reflects throughput/time efficiency (inpatient days relative to discharges). These indicators are commonly used in hospital efficiency measurement as process/throughput metrics and key performance indicators [32,33,34].

Since geography and location impact embeddedness and strategy, we incorporated five environmental or market factors that are commonly cited in the literature as meaningful contributors to hospital financial performance, including the obesity percentage in the surrounding market (population disease burden), median income within the zip code (socioeconomic status), average age of the population, the percentage of the market without health insurance, and total population of the surrounding zip code. All of these variables were measured at the zip code level.

To assess the relationships among financial and structural indicators before regression modeling, we conducted pairwise Pearson correlation analyses using the cleaned analytic dataset. Variance inflation factors (VIFs) were subsequently examined to confirm that no variables exhibited problematic multicollinearity, ensuring the stability and interpretability of the regression estimates.

We estimated logistic regression models with severe financial distress (Z ≤ 1.8; 1 = distressed, 0 = not distressed) as the dependent variable and included hospital structural characteristics (beds, ownership/control type, and urban/rural), operational capacity indicators (occupancy, ALOS), financial structure (debt-to-equity), and ZIP-level market characteristics (income, obesity prevalence, average age, uninsured prevalence, population). Fiscal-year indicator variables were included to account for temporal differences across FY2021–FY2023.

$$>logit\left(P\left({Distress}_{it}=1\right)\right)=\beta_{0}+{Χ}_{it}\beta +M_{zt}\theta +\gamma_{t}>$$

where i indexes hospitals, t fiscal year, and z ZIP code; X includes beds, control type, urban/rural, occupancy, ALOS, and debt-to-equity; M includes ZIP-level income, obesity prevalence, average age, uninsured prevalence, and population; and $\gamma_{t}$ are fiscal-year indicators.

Statistical significance of predictors was assessed using Wald chi-square tests with a significance threshold of *p* < 0.05. Independent and control variables include all the variables described previously. Results are reported as regression coefficients and odds ratios. All analyses were conducted in SPSS (IBM, version 30). Since this study uses publicly available data, this study was exempt from the Institutional Review Board human subject’s protection review.

## 3. Results

There was a total of 7900 short-term acute care hospital observations from fiscal years 2021 through 2023 (2690 in 2021, 2642 in 2022, and 2568 in 2023), representing 2828 unique hospitals. Amongst the 2828 unique hospitals, 83.3% contributed observations in all three years, while 11.0% contributed to two years and 5.7% contributed to one year. Approximately 61% of facilities were classified as urban, and 39% rural. Most hospitals were classified as voluntary non-profit hospitals. Table 1 presents the descriptive statistics for the independent variables by fiscal year.

The mean Z-score varied annually, decreasing from 5.46 in 2021 to 5.37 in 2023. Operating margin (%) similarly reduced from −4.22% to −6.54% and return on assets also declined. Table 2 presents the key metrics for financial distress and performance.

Approximately 18.6% of hospitals were below the 1.8 threshold for severe financial distress in 2021, rising to 21.9% in 2022 and 22.0% in 2023. In 2023, this equates to 566 hospitals exhibiting significant financial distress. Hospital debt burdens increased significantly during this time, from a mean of $816,000 per bed to $1,079,161, an increase of nearly 32%. At the same time, average net patient revenues grew only 13.6%, from $343.3 million to $390.0 million.

After entry into a logistic regression model, with binary outcome 1 indicating financial distress (with a Z-score of <1.8), several characteristics were significant. Table 3 presents the regression model.

Certain structural factors emerged as significant. Larger, urban hospitals, and those with higher average lengths of stay, were significantly more likely to be in distress than smaller, rural hospitals. Those organizations with larger debt-to-equity ratios and lower occupancy rates were more likely to be distressed, suggesting that efficiency (occupancy rate and ALOS) and debt burden play a pivotal role in performance. The surrounding environment appeared to have less influence on distress. Only the population, or the size of the surrounding market, was significant. Hospitals in larger populations were 1.6x more likely to be distressed than hospitals in smaller communities (β = 0.475, *p* < 0.001). The other measures of the environment were not statistically significant when adjusting for multiple factors.

## 4. Discussion

In this national longitudinal analysis of all U.S. short-term acute care hospitals from 2021 to 2023, we observed a meaningful increase in the proportion of hospitals experiencing severe financial distress, rising from 18.6% in 2021 to 22.0% in 2023. Although mean Altman Z-scores did not change significantly across the study period, both operating margins and returns on assets declined substantially, indicating persistent and widespread financial pressure. These results suggest that financial strain within the sector is becoming more concentrated among a sizable subset of hospitals, even as average system-level performance remains relatively stable. These findings align with evidence that financial vulnerability is not evenly distributed across hospitals. Observed performance during and after the pandemic reflects both underlying operational conditions and external funding/policy environments [13].

Operational indicators such as occupancy and length of stay can be interpreted as proxies for capacity utilization and throughput. These reflect the capability of translating fixed resources into delivered inpatient care. In prior US work using Altman Z-based outcomes, operational and structural factors have been associated with distress risk [35]. Our findings demonstrate that hospitals with longer average lengths of stay were more likely to be distressed, whereas higher occupancy rates were protective, highlighting the role of throughput efficiency, capacity management, and care coordination in financial sustainability. Our findings underscore that capital structure matters: highly leveraged organizations may have reduced flexibility to absorb shocks, invest or adapt service lines when costs rise and revenues are low.

The observed structural and operational associations are consistent with frameworks that suggest hospitals are embedded in and constrained by resources that shape demand, workforce costs, and payer composition. From an institutional perspective, hospitals operate under coercive pressures (rules and regulations), normative pressures (accreditation and professional standards), and mimetic pressures (benchmarking against other hospitals, quality metrics and competition under uncertainty), which influence service configuration and strategic flexibility [36]. These pressures are particularly salient in periods of rapid policy change and macroeconomic disruption, as seen in pandemic-era funding and subsequent labor and inflation pressures [13]. These pressures alter operating conditions unevenly particular to our findings in larger hospitals in large metropolitan systems.

Though these findings diverge from earlier literature that emphasizes the vulnerability of rural providers. Our findings may reflect heightened exposure to competitive pressures, higher labor and capital costs, or structural challenges unique to large metropolitan systems.

In large urban markets, community sociodemographic and environmental factors play a more limited role once organizational characteristics are accounted for, which is consistent with Ozcan and Luke (1993) and Zwanziger et al. (2010) [37,38]. Among the market variables included, only population size was significantly associated with distress, with hospitals in larger markets facing greater risk. Median income, obesity prevalence, age distribution, and uninsured rates were not significant predictors in adjusted models, suggesting that local socioeconomic conditions may exert weaker independent effects than previously reported when examined alongside financial and operational indicators.

Taken together, these findings reflect a hospital sector continuing to adapt to post-pandemic recovery challenges, rising operating costs, and shifting patterns of utilization. The interaction between operational performance, capital structure, and market size appears increasingly central in determining financial resilience. Although causal inference is not possible in this observational framework, the associations identified point to several actionable levers for hospital leaders and policymakers. These include improving throughput efficiency, carefully managing debt burdens, and recognizing the heightened risks faced by hospitals operating in large and complex markets. Strengthening these areas is essential to maintaining financial viability and ensuring continued access to acute care services across diverse communities.

Future research should examine how hospital financial distress responds to specific policy and reimbursement changes, including Medicaid reforms and value-based payment initiatives, to better understand causal pathways. Research could also apply sensitivity analyses to assess differences when adjusting the Z-score cutoff values. Additional studies linking financial distress to organizational responses such as service line reductions, workforce changes, consolidation, and closure would further inform policy and practice. Greater use of longitudinal designs with more precise market definitions could also clarify how competition and system affiliation influence hospital financial resilience over time.

### 4.1. Policy and Practice Implications

The policy landscape in the U.S. has become complicated for hospital sustainability. The One Big Beautiful Bill Act (OBBBA), signed into law in 2025, includes over $900 billion in Medicaid cuts over a decade [39].

Over 90% of all hospitals rely on Medicaid, with rural hospitals relying even more on these funds. Reductions are expected to result in 7.6 to 14.4 million Americans losing coverage, disproportionately affecting rural and safety-net hospitals [40]. The Commonwealth Fund projects that further reductions in Medicaid revenue and increases in uncompensated care could force hospitals to reduce costs by eliminating services [41]. Furthermore, hospitals under financial pressure often cut staffing, reduce programs, delay equipment upgrades, or postpone facility improvements, all of which impact quality of care and patient outcomes well before any closures occur. Understanding financial distress helps policymakers diagnose and address systemic failures.

From a practice perspective, hospital administrators should focus on the controllable aspects of their strategy, while also considering the surrounding geographic location for new service line introductions. Hospital financial distress at this scale signals a growing risk of service reductions and closures that could undermine regional healthcare capacity and limit access in many communities

It is vital that hospitals find mechanisms to survive financial distress. The negative impact of hospital closures, including reduced availability of care, fewer services, and shorter service duration, raises significant concerns for individuals seeking quality care.

### 4.2. Limitations

This study is not without its limitations. Although the study is longitudinal and not simply a cross-sectional analysis, the quality of the underlying data is always a major concern. This study relies on data extracted from the Medicare cost reports. While all attempts were made to ensure the data were valid, all coded data has inherent limitations. We applied statistical tools to address null and extreme values, but secondary data quality is always a concern. Second, the Z-score model itself is not specifically calibrated for hospitals. It has been applied to healthcare and many other service industries, but there may be differences in how scores are interpreted when different parameters or thresholds are used. Third, we did not measure changes in the number and breadth of hospital service lines, which could impact performance. The dataset does not include direct measures of closure, bankruptcy, or service suspension. As a result, hospitals that exit the market during FY2021-FY2023 are represented only in years with available cost reports, and estimates should be interpreted as associations among observed hospital-year records rather than causal effects of closure dynamics. Future research could build on these limitations by using other structural and geographic measures, or linking cost report panels with external closure and consolidation datasets in their model.

## 5. Conclusions

This study contributes national, post-pandemic evidence that financial distress remains widespread and is increasingly concentrated among U.S. acute care hospitals, underscoring the fragility of hospital financial sustainability in the current policy and economic environment. By integrating standardized financial distress measures with organizational and market characteristics, the analysis highlights operational efficiency and capital structure—rather than local sociodemographic conditions—as central drivers of financial vulnerability, particularly in large and complex urban markets.

From a policy and practice perspective, these findings suggest that strategies to strengthen hospital resilience should prioritize improvements in throughput efficiency, capacity utilization, and prudent debt management, alongside targeted oversight and support for hospitals operating in high-cost, competitive markets. As hospitals continue to face reimbursement pressures, labor shortages, and potential reductions in public funding, timely identification of financial distress can inform interventions aimed at preserving access to essential acute care services and preventing destabilizing service reductions or closures.

## Figures and Tables

**Table 1:** Descriptive characteristics.

Characteristic	FY2021 (n = 2690)	FY2022 (n = 2642)	FY2023 (n = 2568)	Total (n = 7900)
Structural				
Size/Beds, mean (SD)	237.562 (237.617)	235.405 (240.218)	234.390 (239.061)	235.810 (238.932)
Control: Governmental, n (%)	383 (14.2%)	379 (14.3%)	383 (14.9%)	1145 (14.5)
Control: Non-Profit, n (%)	1727 (64.2%)	1690 (64.0%)	1626 (63.3%)	5043 (63.8)
Control: Proprietary, n (%)	580 (21.6%)	573 (21.7%)	559 (21.8%)	1712 (21.7)
Urban, n (%)	1673 (62.2%)	1615 (61.1%)	1515 (59.0%)	4803 (60.8)
Rural, n (%)	1017 (37.8%)	1027 (38.9%)	1053 (41.0%)	3097 (39.2)
Operational				
Average length of stay, mean (SD)	4.748 (1.965)	4.801 (2.052)	5.620 (50.334)	5.049 (28.747)
Occupancy Rate, mean (SD)	0.497 (0.235)	0.505 (0.233)	0.491 (0.236)	0.498 (0.234)
Debt/Equity ratio, mean (SD)	4.807 (68.123)	3.738 (24.981)	6.106 (61.559)	4.871 (54.962)
Environmental/Market				
Median Income, $ (SD)	39,247 (14,196)	39,484 (14,242)	39,466 (14,238)	39,397 (14,223)
Obesity % (SD)	34.929 (6.512)	34.866 (6.530)	34.905 (6.532)	34.899 (6.52)
Average Age, years (SD)	39.110 (6.205)	39.071 (6.149)	39.069 (6.158)	39.080 (6.170)
% Uninsured (SD)	12.475 (9.374)	12.398 (9.293)	12.418 (9.412)	12. 41 (9.41)
Population (per 100,000), mean (SD)	0.301 (0.173)	0.301 (0.174)	0.303 (0.174)	0.302 (0.174)

Unit of analysis is the hospital’s year; hospitals may contribute from 1 to 3 years of observations.

**Table 2:** Key measures of financial distress.

Measure	2021	2022	2023	*p*-Value
Z-score	5.46 (4.786)	5.33 (5.095)	5.37 (5.232)	*p* = 0.886
Operating Margin	−4.22 (66.755)	−7.76 (40.465)	−6.55 (29.650)	*p* < 0.05
Return on Assets	10.85 (20.662)	3.00 (22.892)	5.05 (21.378)	*p* < 0.001

**Table 3:** Logistic regression, odds of severe financial distress.

Variable	B	S.E.	Wald	OR	95% C.I. for EXP(B)			
					Lower	Upper	*p*-Value	Sig
Beds (size)	0.000	0.000	6.107	1.000	0.999	1.000	0.013	*
Type (control)	0.031	0.039	0.632	1.032	0.955	1.114	0.427	
Urban	0.237	0.052	21.084	1.268	1.146	1.402	<0.001	**
ALOS	0.063	0.014	21.062	1.065	1.037	1.094	<0.001	**
Occupancy Rate	−1.157	0.118	96.245	0.314	0.249	0.396	<0.001	**
Debt-Equity	0.050	0.003	261.308	1.051	1.045	1.057	<0.001	**
Median Income	−0.004	0.004	1.145	0.996	0.988	1.004	0.285	
Obesity	−0.007	0.004	3.065	0.993	0.986	1.001	0.080	
Age	0.004	0.004	0.904	1.004	0.996	1.013	0.342	
% Uninsured	−0.372	0.339	1.207	0.689	0.355	1.339	0.272	
Population	0.475	0.146	10.580	1.609	1.208	2.143	0.001	**
Constant	−1.439	0.289	24.793	0.237			<0.001	**

* *p* < 0.05. ** *p* = <0.001. R^2^ = 0.097, χ^2^ = 808.46, df = 11, *p* < 0.001. n = 7900. OR = odds ratio.

## Data Availability

Restrictions apply to the availability of these data. Data were obtained from American Hospital Directory and US Census Bureau and are available at https://www.ahd.com/ and https://census.gov with the permission of American Hospital Directory and US Census Bureau.

## Abbreviations

The following abbreviations are used in this manuscript:

ALOS	Average length of stay
OR	Odds ratio
ROA	Return on assets
